# Novel Antioxidants and *α*-Glycosidase and Protein Tyrosine Phosphatase 1B Inhibitors from an Endophytic Fungus *Penicillium brefeldianum* F4a

**DOI:** 10.3390/jof7110913

**Published:** 2021-10-27

**Authors:** Yan Bai, Ping Yi, Songya Zhang, Jiangchun Hu, Huaqi Pan

**Affiliations:** 1 Institute of Applied Ecology, Chinese Academy of Sciences, Shenyang 110016, China; baiy@iae.ac.cn (Y.B.); hujc@iae.ac.cn (J.H.); 2 The Key Laboratory of Chemistry for Natural Product of Guizhou Province and Chinese Academy of Sciences, Guiyang 550002, China; yiping@gzcnp.cn; 3 CAS Key Laboratory of Quantitative Engineering Biology, Shenzhen Institute of Synthetic Biology, Shenzhen Institute of Advanced Technology, Chinese Academy of Sciences, Shenzhen 518055, China; sy.zhang2@siat.ac.cn

**Keywords:** endophyte, peniorcinol, DPPH^•^ scavenging activity, ABTS^•+^ scavenging activity, *α*-glycosidase inhibitory activity, protein tyrosine phosphatase 1B (PTP1B) inhibitory activity

## Abstract

Oxidative stress plays a very important role in the progression of diabetes and its complications. A therapeutic agent that is both antidiabetic and antioxidant would be the preferred choice for the treatment of diabetes. The crude extract of the endophytic fungus *Penicillium brefeldianum* F4a has significant antioxidant and *α*-glycosidase and protein tyrosine phosphatase 1B (PTP1B) inhibition activities. Chemical investigation of *P. brefeldianum* F4a using an activity-guided isolation led to the discovery of three new compounds called peniorcinols A–C (**1**–**3**) along with six known compounds: penialidins A (**4**), penialidin F (**5**), myxotrichin C (**6**), riboflavin (**7**), indole-3-acetic acid (**8**), and 2-(4-hydroxy-2-methoxy-6-methylphenyl) acetic acid (**9**). Their chemical structures were established by their NMR and HRESIMS. The absolute configurations of **1** and **3** were determined by experimental and calculated electronic circular dichroism (ECD). Their antioxidant activities were evaluated by DPPH^•^ and ABTS^•+^ scavenging assays. Compounds **1**–**6** and **8**–**9** showed moderate to strong free radical scavenging activities. Significantly, **4**–**6** exhibited more potent ABTS^•+^ scavenging activity than that of the positive control. Their *α*-glycosidase and PTP1B inhibition activities were tested. Among them, compound **3** showed α-glucosidase inhibition activity, and compounds **7** and **8** showed PTP1B inhibitory activity for the first time. It is worth noting that **3** and **8** displayed both antioxidant and *α*-glycosidase or PTP1B inhibition activities. These finding suggest that compounds **3** and **8** could be used as lead compounds to generate new potent drugs for the treatment of oxidative stress-related diabetes.

## 1. Introduction

Reactive oxygen species are produced by living organisms because of normal cellular metabolism and environmental factors, which can damage cell structures and alter their functions [1]. The balance between oxidant and antioxidant in favor of oxidants is termed “oxidative stress” [2], which may cause many diseases, including diabetes mellitus [3] and cancer [4]. Notably, much evidence indicates that oxidative stress plays a very important role in the progression of diabetes and its complications [3,5,6], for instance, diabetic nephropathy, cardiomyopathy, and diabetic neuropathy [7,8]. In addition, uncontrolled chronic hyperglycemia is directly related to free radical overload [9]. Therefore, a therapeutic agent that possesses both antioxidants and antidiabetics would be the preferred choice for diabetes [7,8].

Endophytic fungi have proven to be an important source of secondary metabolites with novel structures and various biological effects [10,11]. The secondary metabolites from endophytic fungi were isolated from two main genera, *Aspergillus* and *Penicillium* [12,13]. *Penicillium* is a widely explored versatile genus due to its chemical diversity and associated biological properties [14,15]. Recently, some novel antidiabetic and antioxidant secondary metabolites isolated from *Penicillium* have been continuously reported. For instance, two new isocoumarins, penicimarins L and M, with antioxidant and *α*-glucosidase inhibitory activities were isolated from fungus *Penicillium* sp. MGP11 [16]. Three xanthones as *α*-glucosidase inhibitors were isolated from an endophytic *Penicillium canescens* [17].

During our ongoing search for natural products with antioxidant and antidiabetic activities from endophytic fungi, the crude extract of *Penicillium brefeldianum* F4a showed significant ABTS^•+^ scavenging activity (EC_50_ = 0.03 mg/mL) and DPPH^•^ scavenging activity (EC_50_ = 0.34 mg/mL), along with *α*-glycosidase inhibition activity (EC_50_ = 0.13 mg/mL) and PTP1B inhibition activity (EC_50_ = 0.03 mg/mL). Therefore, in order to investigate dual-active secondary metabolites with antioxidant and potential antidiabetic activities from the endophyte strain F4a, three new compounds, peniorcinols A–C (**1**–**3**), and six known ones (**4**–**9**) were isolated. Herein, we outline their isolation and structure elucidation, together with antioxidant and *α*-glycosidase and PTP1B inhibition activities.

## 2. Materials and Methods

### 2.1. General Experimental Procedures

Semipreparative HPLC was recorded using a Dionex UltiMate 3000 HPLC system equipped with multiple wavelength detectors utilizing YMC-Pack-ODS-A column (250 × 10 mm, 5 μm). Optical rotations were acquired by using an SGW-2 digital polarimeter. IR spectra were measured with a Thermo fisher Nicolet 6700 FT-IR spectrometer in KBr discs. HRESIMS data were obtained on a Thermo Scientific Q Exactive mass spectrometer. ^1^H NMR (600 MHz), ^13^C NMR (150 MHz), and 2D NMR were tested by a Bruker-AV-600 NMR spectrometer using solvent signals as references. The ECD spectra were measured using a Science MOS-450 spectrometer. Column chromatography was performed with silica gel (200–300 mesh; Qingdao Ocean Chemical Co. Ltd., Qingdao, China), Sephadex LH-20 gel (GE Healthcare, Uppsala, Sweden), and ODS-A reversed-phase silica gel (50 μm; YMC Co. Ltd., Kyoto, Japan). The supernatant was treated with macroporous resin HP20 (Mitsubishi Chemical Co. Ltd., Kyoto, Japan).

### 2.2. Fungal Material

The fungus F4a was isolated from the roots of *H. cordata* and identified as *P. brefeldianum* (a former synonym of *Eupenicillium brefeldianum*) [18], and it has been stored at the Institute of Applied Ecology, Chinese Academy of Sciences.

### 2.3. Fermentation and Extraction

The fungus F4a was incubated on a culture medium (0.25% barley extract, 0.1% yeast extract, 0.05% soybean cake, 2% glucose, 1% starch, 0.4% CaCO_3_, 0.3% KH_2_PO_4_, 0.25% MgSO_4_·7H_2_O, 0.1% (NH_4_)_2_SO_4_, 0.08% KNO_3_, and 0.002% CuSO_4_, pH = 7.0) at 28 °C for 7 days at 180 rpm. The fermentation broth was centrifuged (4000 rpm for 20 min) to obtain supernatant and mycelium. The supernatant was treated with macroporous resin HP20 and stirred for 2 h (28 °C, 180 rpm). The resin was then filtered and eluted three times with methanol. The methanol was collected and dried by a rotary evaporator to obtain extract A. The mycelium was extracted three times by acetone and put in an ultrasonic water bath for 40 min. The acetone was collected and dried by a rotary evaporator to obtain extract B. A total of 35 g was obtained by combining extracts A and B.

### 2.4. Bioactivity-Guided Isolation

The extract was chromatographed by a silica gel column (350 g, 5 × 100 cm) with CH_2_Cl_2_-CH_3_OH (100:0, 100:1, 100:2, 100:5, 100:7, 100:10, 100:20, 100:30, and 100:50, *v*/*v*) to partition nine fractions (A–I), and the bioactivities of each fraction were measured. The target fraction B was separated by Sephadex LH-20 (2 × 150 cm, MeOH) to yield two subfractions (B_1_ and B_2_). The target subfraction B_1_ was further purified by MeOH recrystallization. The target subfraction B_2_ was subjected to ODS (50 μm, 3.5 × 40 cm) eluting with MeOH-H_2_O (gradient 30:70, 35:65, 40:60, 45:55, 50:50, 55:45, and 60:40, *v*/*v*). The target fraction C was separated by Sephadex LH-20 (2 × 150 cm, MeOH) to yield two subfractions (C_1_ and C_2_). The target subfraction C_1_ was subjected to semipreparative HPLC (MeOH-0.05% trifluoroacetic acid H_2_O, 55:45, *v*/*v*) at a flow rate of 2.5 mL/min at 35 °C, and the subfraction C_2_ was subjected to ODS (50 μm, 3.5 × 40 cm) eluting with a MeOH-H_2_O (gradient 30:70, 35:65, 40:60, 45:55, 50:50, 55:45, and 60:40, *v*/*v*), followed by semipreparative HPLC (MeOH-0.05% trifluoroacetic acid H_2_O, 45:55, *v*/*v*) at a flow rate of 2.5 mL/min at 35 °C. The target fraction E was further purified by MeOH recrystallization.

### 2.5. Spectral Data

*Peniorcinol A* (**1**): colorless, transparent oil, ${\left[\alpha \right]}_{D}^{27}$-33 (*c* 0.55, MeOH); HRESIMS *m*/*z* 319.1190 [M-H]^−^ (calcd for C_17_H_19_O_6_, 319.1182) (Figure S1); ECD (*c* 0.025, MeOH) *λ*_max_ (Δε) 214 (−0.85), 224 (+1.36), 235 (−0.16), 254 (+1.50) nm; IR (KBr) *ν*_max_ 3706, 2972, 1677, 1577, 1334, 1198, 1142, 1012 cm^−1^ (Figure S2); ^1^H and ^13^C NMR data, Table 1, Figures S3 and S4.

*Peniorcinol B* (**2**): white powder, HRESIMS *m*/*z* 277.1068 [M + H]^+^ (calcd for C_15_H_17_O_5_, 277.1076) (Figure S8); IR (KBr) *ν*_max_ 3705, 2974, 1683, 1577, 1339, 1012 cm^−1^ (Figure S9); ^1^H and ^13^C NMR data, Table 1, Figures S10 and S11.

*Peniorcinol C* (**3**): Colorless, transparent oil; ${\left[\alpha \right]}_{D}^{25}$ + 35 (*c* 0.1, MeOH); HRESIMS *m*/*z* 297.1100 [M + Na]^+^ (calcd for C_16_H_18_O_4_Na, 297.1100) (Figure S14); ECD (*c* 0.025, MeOH) *λ*_max_ (Δε) 203 (+9.95), 230 (−0.39), 236 (+0.01), 245 (−0.49), 255 (−0.19), 285 (−1.65), 303 (−0.34) nm; IR (KBr) *ν*_max_ 3397, 2938, 1683, 1602, 1200, 1143, 1059, 1031 cm^−1^ (Figure S15); ^1^H and ^13^C NMR data, Table 1, Figures S16 and S17.

### 2.6. ECD Calculations

The CONFLEX analyses of compounds **1** and **3** were performed with Merck Molecular Force Field (MMFF). Subsequently, the conformations that showed >1% Boltzmann population were subjected to geometry optimization by density functional theory (DFT) at the B3LYP/6-311+G (d, 2p) level. Then, the time-dependent DFT (TDDFT) method at the CAM-B3LYP/TZVP level was used on the lowest energy conformations for each configuration with a PCM model in a methanol solution [19]. Finally, the overall theoretical ECD curves were simulated using SpecDis 1.71 software (University of Wurzburg, Wurzburg, Germany) on the basis of the Boltzmann weighting of each conformer [20]. All calculations were performed with the Gaussian 09 program package [21].

### 2.7. Antioxidant Assay

#### 2.7.1. DPPH Radical Scavenging Assay

The DPPH free radicals scavenging assay was tested according to the previously reported methods [22]. First, 100 μL of sample at different concentrations in ethanol (the blank control used 100 μL of ethanol instead of the sample) was added to 96 wells, followed by 100 μL of 0.15 mM DPPH. After incubating for 30 min in darkness at room temperature, the reduction of DPPH radical was determined by measuring the absorbance at 517 nm with a microplate reader. *L*-Ascorbic acid was used as the positive control.

#### 2.7.2. ABTS Radical Scavenging Assay

The ABTS free radical scavenging assay was tested according to the previously reported methods [23]. The ABTS radical cation solution was prepared by mixing 2.45 mM potassium persulphate and 7 mM ABTS (1:1, *v*/*v*). The mixture was incubated in darkness at room temperature for 12 h before use. The ABTS cation radical solution was diluted with water to obtain an absorbance of 0.70 ± 0.02 at 734 nm. Then, 100 μL of the sample at various concentrations in ethanol (the blank control used 100 μL of ethanol instead of the sample) was added to 3.0 mL of diluted ABTS^•+^ solution and mixed vigorously. After incubating for 6 min in darkness at room temperature, the absorbance was measured at 734 nm with a microplate reader. *L*-Ascorbic acid was used as the positive control.

### 2.8. Antidiabetic Assay In Vitro

#### 2.8.1. α-Glycosidase Inhibitory Assay

The *α*-glucosidase inhibitory assay was determined by the method described by Apostolidis and Lee with slight modifications [24]. First, 30 μL of the 2 U/mL *α*-glycosidase in 0.1 M phosphate buffer solution (PBS) and 20 μL of the sample at different concentrations in DMSO (the blank control used 20 μL of DMSO instead of the sample) were mixed in 800 μL of 0.1 M PBS (pH 6.8), incubating for 5 min at 37 °C in a water bath. After preincubation, 150 μL of 10 mM *p*-nitrophenyl glucopyranoside (*p*-NPG) was added to the mixture to initiate the reaction. After reacting for 30 min, 650 μL of 1 M Na_2_CO_3_ solution was added to terminate the reaction. The enzyme activity was determined by measuring the absorbance of *p*-nitro phenol at 405 nm with a microplate reader. Acarbose was used as the positive control.

#### 2.8.2. PTP1B Inhibitory Assay

The PTP1B inhibitory activity was established according to the previously reported methods [25]. The reagent para-nitrophenyl phosphate (*p*-NPP) was used as a substrate for PTP1B. Then, 81 μL enzyme in buffer (pH 7.5), including EDTA (1 mM), Tris-HCl (25 mM), dithiothreitol (DTT) (1 mM), *β*-mercaptoethanol (2 mM), and 10 μL of sample at various concentrations in DMSO (the blank control used 10 μL of DMSO instead of the sample), was incubated in 96-well plates at 37 °C for 10 min. Then, 4 μL *p*-NPP (10 mM) in buffer was added and incubated at 37 °C for 30 min. Next, 5 μL of 2 M NaOH solution was added to terminate the reaction. Then, the absorbance was determined at 405 nm wavelength. Sodium orthovanadate (Na_3_VO_4_) was used as the positive control.

## 3. Results and Discussion

### 3.1. Bioactivity-Guided Compound Isolation

**Figure 1:**
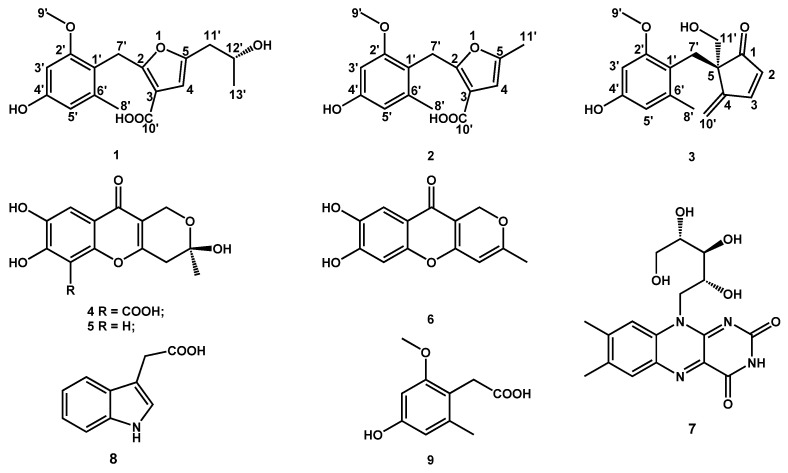
Chemical structures of compounds **1**–**9.**

The active extract of the strain *P. brefeldianum* F4a showed significant DPPH^•^ and ABTS^•+^ scavenging activities, together with *α*-glycosidase and PTP1B inhibition activities. Then, the extract was further isolated by silica gel column chromatography to afford nine fractions (A–I), and fractions B and C, together with E, showed bioactivity. Fraction B (4.5 g) displayed significant ABTS^•+^ scavenging and PTP1B inhibition activities, fraction C (2.2 g) showed antioxidant activity, and fraction E (0.5 g) exhibited significant PTP1B inhibition activity, suggesting that the strain F4a can not only produce secondary metabolites with antioxidant or *α*-glycosidase and PTP1B inhibition activities but also may produce secondary metabolites with the above two kinds of activities. Compound **8** (3.4 mg) was recrystallized from ABTS^•+^ scavenging and PTP1B inhibition activities subfraction B_1_ (0.1 g). Compounds **2** (135.1 mg), **3** (16.6 mg), and **4** (313.2 mg) were purified and fractionated by ODS and semipreparative HPLC from antioxidant subfraction B_2_ (2.5 g). Compounds **1** (27.6 mg), **5** (34.0 mg), **6** (3.6 mg), and **9** (17.3 mg) were purified from the antioxidant fraction C by Sephadex LH-20 and semipreparative HPLC. Compound **7** (10.0 mg) was isolated from fractions E (0.5 g). Finally, all of the monomer compounds were systematically evaluated for four biological activity assays. Although compound **3** was isolated from the antioxidant subfraction B_2_, compound **3** exhibited weak *α*-glycosidase inhibition activity (Table 2). This could be because the content of compound **3** is relatively low, resulting in the *α*-glycosidase inhibition activity of subfractions B_2_ not being detected. The chemical structures of compounds **1**–**9** are shown in Figure 1.

### 3.2. Structure Elucidation

**Figure 2:**
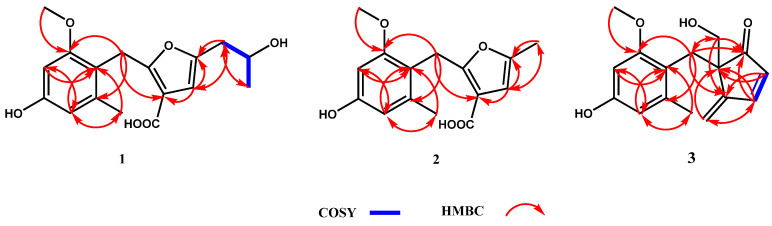
Key ^1^H-^1^H COSY and HMBC correlations of compounds **1**–**3.**

**Figure 3:**
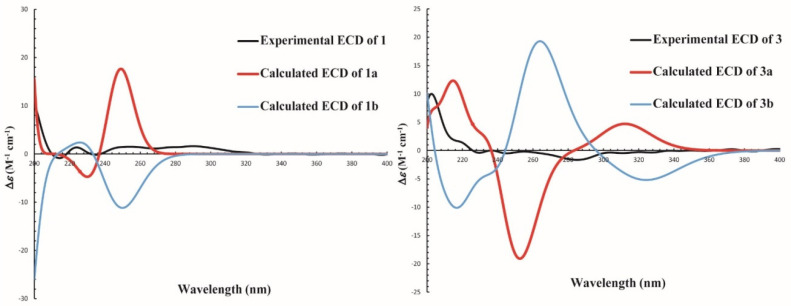
Experimental ECD and calculated ECD spectra of compounds **1** and **3** in CH_3_OH.

The molecular formula of compound **1** was determined as C_17_H_20_O_6_ by HRESIMS analysis at 319.1190 [M-H]^−^ (calcd for C_17_H_19_O_6_, 319.1182). The 1D NMR of **1** indicated one OCH_3_, one doublet CH_3_, one singlet CH_3_, one singlet CH_2_, four CHs (three olefinic bonds), and eight quaternary carbons (Table 1). Six signals in ^13^C NMR (*δ*_C_ 158.6, 155.7, 138.1, 116.9, 108.9, and 96.8) were annotated to a tetra-substituted benzene, and the positions of substitutes were determined by the HMBC correlations of H-3′/C-1′, C-5′; H-5′/C-1′, C-3′, C-8′; H-9′/C-2′; H-8′/C-1′, C-5′; and H-7′/C-2′, C-6′ (Figure 2, Figures S5 and S6). The structure of a 2, 3, 5-tri-substituted furanic acid was established by four signals in ^13^C NMR (*δ*_C_ 164.6, 160.4, 100.9, and 100.8) and the HMBC correlations of H-4/C-3, C-5, C-10′, C-11′; H-11′/C-4, C-5; H-7′/C-3 (Figure 2, Figures S5 and S6). The fragment of a 2-hydroxy-propyl group was established by the COSY correlations (H-11/H-12/H-13) (Figure 2 and Figure S7). Finally, the connections of these fragments were determined from the HMBC correlations of H-7′/C-3, C-2′, C-6′ and H-11′/C-4, C-5 (Figure 2, Figures S5 and S6). To determine the absolute configuration of **1**, the optimized conformations of (12′*R*)-**1**a and (12′*S*)-**1**b were obtained at the B3LYP/6-311+G (d, 2p) level and used for ECD calculations. The calculated ECD curve showed a high degree of similarity to the experimental ECD spectrum of **1**a (Figure 3), which indicated the absolute configuration of **1** is 12′*R*. Therefore, compound **1** was characterized as a new skeleton compound with orcinol structure and named peniorcinol A.

Compound **2** was obtained as a white powder with the molecular formula of C_15_H_16_O_5_ by HRESIMS *m*/*z* 277.1068 [M + H]^+^ (calcd for C_15_H_17_O_5_, 277.1076). The 1D NMR spectra of compound **2** indicated a similar skeleton to compound **1** in addition to the substituent group of C-5 (Table 1). The data of 1D NMR (*δ*_H_ 2.09, s and *δ*_C_ 19.2, CH_3_), HRESIMS, and the HMBC correlations of H-11′/C-4, C-5 revealed that the 2-hydroxy-propyl group of **1** was replaced by a methyl in **2** (Table 1, Figure 2, Figures S12 and S13). Therefore, compound **2** was characterized as a new derivative of **1** and named peniorcinol B.

Compound **3** was obtained as a colorless and transparent oil with the molecular formulas of C_16_H_18_O_4_ by HRESIMS *m*/*z* 297.1100 [M + Na]^+^ (calcd for C_16_H_18_O_4_Na, 297.1100). The first fragment of 4-hydroxy-2-methoxy-6-methylbenzyl was deduced by comparison of its 1D NMR with **1** (Table 1). The second fragment of 5-(hydroxymethyl)-4-methylenecyclopentanone was deduced by the HMBC correlations of H-2/C-4, C-5; H-3/C-1, C-5, C-10′; H-10′/C-3, C-5; and H-11′/C-1, C-4, C-7′ (Figure 2 and Figures S18–S20). The carbonyl at C-1 was established by the HMBC correlations of C-1/H-3, H-7′, H-11′ (Figure 2). In addition, C-7′ connected to C-5 and C-1′ was established by the HMBC of H-7′/C-1, C-4, C-2′, C-6′, C-11′ (Figure 2 and Figures S18–S20). Furthermore, the absolute configuration of **3** was determined by ECD analysis and the calculated ECD curve showed a high degree of similarity to the experimental ECD spectrum of **3**a (Figure 3), which indicated that the absolute configuration of **3** is 5*R*. Therefore, compound **3** was characterized as a new skeleton compound and named peniorcinol C.

Six known compounds were identified as penialidin A (**4**), penialidin F (**5**), myxotrichin C (**6**), riboflavin (**7**), indole-3-acetic acid (**8**), and 2-(4-hydroxy-2-methoxy-6-methylphenyl) acetic acid (**9**) by comparing their spectroscopic data with the literature (Figure 1) [26,27,28,29,30,31].

### 3.3. Antioxidant Activities

The antioxidant activities of compounds **1**–**9** were assessed using two methods: DPPH and ABTS. In the DPPH^•^ assay (Table 2), compounds **5** and **6** showed moderate activity (EC_50_ = 28.42 and 30.07 μM) compared with the positive control (EC_50_ = 6.12 μM). To the best of our knowledge, this is the first report of DPPH^•^ scavenging activities of compounds **5** and **6**. The DPPH^•^ scavenging activities of **4** have been reported in previous studies (IC_50_ = 156.38 μg/mL) [32], which is in agreement with our results (EC_50_ > 100 μM). Compared with the same skeleton of chromone (**4**–**6**), compound **4** (EC_50_ > 100 μM) was found to be less active in DPPH^•^ scavenging than compounds **5** and **6** (EC_50_ = 28.42 and 30.07 μM), suggesting that the carboxyl group (-COOH) significantly reduced the DPPH^•^ scavenging activity.

In the ABTS^•+^ assay (Table 2), compounds **1**–**6** and **8**–**9** showed ABTS^•+^ scavenging activity (EC_50_ values ranging from 7.61 to 32.67 μM), which is the first report of their ABTS^•+^ scavenging activities. Significantly, compounds **4**–**6** exhibited significant ABTS^•+^ scavenging activities (EC_50_ = 14.54, 7.61, and 14.96 μM), which were more potent than that of the positive control (EC_50_ = 18.81 μM). These results illustrated that chromones (**4**–**6**) showed stronger ABTS^•+^ scavenging activity than that of the other compounds (**1**–**3** and **7**–**9**), which is consistent with the DPPH^•^ scavenging activity. These results can support the potential of compounds **4**–**6** to be explored as potential natural antioxidant agents.

### 3.4. α-Glycosidase and PTP1B Inhibition Activities

All of the compounds were evaluated for antidiabetic activities by *α*-glycosidase and PTP1B inhibition assays (Table 2). *α*-Glycosidase inhibitors delay complex carbohydrate digestion and reduce carbohydrate absorption into monosaccharides, thereby reducing blood glucose levels [33]. New compound **3** showed weak *α*-glucosidase inhibition activity (EC_50_ = 38.93 μM) compared with the positive control (EC_50_ = 2.62 μM). In contrast, orcinol analogs **1**, **2**, and **9** had no *α*-glycosidase inhibition activity (EC_50_ > 50 μM), suggesting that the fragment of 5-(hydroxymethyl)-4-methylenecyclopentanone of compound **3** might be a potent pharmacophore for the discovery of *α*-glycosidase inhibitors.

Since the discovery of PTP1B in 1988, it has become a highly plausible candidate for therapeutic inhibitors of obesity and type 2 diabetes [34]. As shown in Table 2, compounds **7** and **8** showed moderate PTP1B inhibitory activity (EC_50_ = 8.87 and 11.68 μM, respectively) compared with the positive control (EC_50_ = 2.38 μM). To the best of our knowledge, this is the first report of their PTP1B inhibitory activities.

There is a large body of evidence demonstrating that oxidative stress plays a very important role in the progression of diabetes and its complications [3,5,6]. Therefore, a therapeutic agent that has both antidiabetic and antioxidant activities would be the preferred choice for diabetes. Compounds **3** and **8** displayed both antioxidant and *α*-glycosidase or PTP1B inhibition activities, which could be explored as potential natural antioxidant and antidiabetic agents for use in oxidative stress-related diabetes. Further, it is necessary to study the antidiabetic activities of these compounds in vivo.

## 4. Conclusions

In summary, three new compounds, peniorcinols A–C (**1**–**3**), and six known compounds (**4**–**9**) were isolated from the endophytic fungus *P. brefeldianum* F4a. In the bioassays, compounds **1**–**6** and **8**–**9** showed moderate to strong radicals scavenging activity. Significantly, compounds **4**–**6** exhibited more potent ABTS^•+^ scavenging activity than that of the positive control. Additionally, compounds **3**, **7**, and **8** showed potential *α*-glycosidase or PTP1B inhibition activities. Compounds **3** and **8** displayed both radical scavenging activity and *α*-glycosidase or PTP1B inhibition activity, which suggests that they could be used as lead compounds to generate potent drugs for the treatment of oxidative stress-related diabetes.

## Figures and Tables

**Table 1:** ^1^H and ^13^C NMR data of **1**–**3** in DMSO-*d*_6_ (*δ* in ppm, *J* in Hz).

Position	1		2		3	
	*δ*_H_	*δ*_C_	*δ*_H_	*δ*_C_	*δ*_H_	*δ*_C_
1						209.0, C
2		164.6, C		164.7, C	6.17, d, (5.6)	132.5, CH
3		100.9, C		100.5, C	7.76, d, (5.6)	158.6, CH
4	5.92, s	100.8, CH	5.89, s	100.0, CH		150.8, C
5		160.4, C		159.3, C		55.7, C
1′		116.9, C		116.9, C		114.6, C
2′		158.6, C		158.6, C		158.8, C
3′	6.14, d, (2.1)	96.8, CH	6.14, d, (1.8)	96.7, CH	6.09, d, (2.2)	96.3, CH
4′		155.7, C		155.7, C		156.2, C
5′	6.09, d, (2.1)	108.9, CH	6.09, d, (1.8)	108.9, CH	6.08, d, (2.2)	108.9, CH
6′		138.1, C		138.1, C		138.4, C
7′	3.45, s	19.6, CH_2_	3.45, s	19.5, CH_2_	2.65, d, (14.0) 2.55, d, (14.0)	28.8, CH_2_
8′	2.16, s	19.9, CH_3_	2.16, s	19.9, CH_3_	2.01, s	20.4, CH_3_
9′	3.60, s	55.3, OCH_3_	3.60, s	55.3, OCH_3_	3.52, s	54.4, OCH_3_
10′	10.76, s	164.3, C	10.74, s	164.2, C	5.23, s 4.96, s	111.9, CH_2_
11′	2.39, m	42.7, CH_2_	2.09, s	19.2, CH_3_	3.63, d, (10.3) 3.55, d, (10.3)	64.7, CH_2_
12′	3.86, m	64.0, CH				
13′	1.06, d, (6.2)	23.4, CH_3_				

**Table 2:** Inhibitory activities on *α*-glycosidase and PTP1B together with antioxidant activity of compounds **1**–**9**.

Compd.	*α*-Glycosidase Inhibitory Assay	PTP1B Inhibitory Assay	DPPH^•^ Assay	ABTS^•+^ Assay
	EC_50_ (μM) ^a^	EC_50_ (μM) ^a^	EC_50_ (μM) ^a^	EC_50_ (μM) ^a^
**1**	>50	>50	>100	21.93 ± 1.06
**2**	>50	>50	>100	28.20 ± 2.87
**3**	38.93 ± 4.90	>50	>100	32.67 ± 3.86
**4**	>50	>50	>100	14.54 ± 0.46
**5**	>50	>50	28.42 ± 3.16	7.61 ± 0.46
**6**	>50	>50	30.07 ± 2.83	14.96 ± 2.57
**7**	>50	8.87 ± 0.91	>100	>100
**8**	>50	11.68 ± 1.03	>100	21.48 ± 0.88
**9**	>50	>50	>100	30.02 ± 4.17
Acarbose	2.62 ± 0.06	ND	ND	ND
Na_3_VO_4_	ND	2.38 ± 0.07	ND	ND
*L*-Ascorbic acid	ND	ND	6.12 ± 0.17	18.81 ± 3.39

ND: not determined. Each value is expressed as a mean ± standard deviation (*n* = 3). ^a^ EC_50_ values correspond to the sample concentration achieving 50% of activity.

## Supplementary Materials

The following are available online at https://www.mdpi.com/article/10.3390/jof7110913/s1. Figure S1: HRESIMS spectrum of compound **1**, Figure S2: IR spectrum of compound **1**, Figure S3: ^1^H NMR (600 MHz, DMSO-*d*_6_) spectrum of compound **1**, Figure S4: ^13^C NMR (150 MHz, DMSO-*d*_6_) spectrum of compound **1**, Figure S5: HSQC spectrum of compound **1**, Figure S6: ^1^H-^13^C HMBC spectrum of compound **1**, Figure S7: ^1^H-^1^H COSY spectrum of compound **1**, Figure S8: HRESIMS spectrum of compound **2**, Figure S9: IR spectrum of compound **2**, Figure S10: ^1^H NMR (600 MHz, DMSO-*d*_6_) spectrum of compound **2**, Figure S11: ^13^C NMR (150 MHz, DMSO-*d*_6_) spectrum of compound **2**, Figure S12: HSQC spectrum of compound **2**, Figure S13: ^1^H-^13^C HMBC spectrum of compound **2**, Figure S14: HRESIMS spectrum of compound **3**, Figure S15: IR spectrum of compound **3**, Figure S16: ^1^H NMR (600 MHz, DMSO-*d*_6_) spectrum of compound **3**, Figure S17: ^13^C NMR (150 MHz, DMSO-*d*_6_) spectrum of compound ^3^, Figure S18: HSQC spectrum of compound **3**, Figure S19: ^1^H-^13^C HMBC spectrum of compound **3**, Figure S20: ^1^H-^1^H COSY spectrum of compound **3**.

## References

[1] Stadtman E.R. (2004). Role of Oxidant Species in Aging. Curr. Med. Chem..

[2] Birben E., Sahiner U.M., Sackesen C., Erzurum S., Kalayci O. (2012). Oxidative stress and antioxidant defense. World Allergy Organ..

[3] Erbağcı M.O., Tuna G., Köse S., Dal-Bekar N.E., Akış M., Kant M., Altunyurt S., Işlekel G.H. (2021). Association between early oxidative DNA damage and iron status in women with gestational diabetes mellitus. Reprod. Toxicol..

[4] Ebadollahi S.H., Pouramir M., Zabihi E., Golpour M., Aghajanpour-Mir M. (2020). The Effect of Arbutin on The Expression of Tumor Suppressor P53, BAX/BCL-2 Ratio and Oxidative Stress Induced by Tert-Butyl Hydroperoxide in Fibroblast and LNcap Cell Lines. Cell J..

[5] Brownlee M. (2005). The Pathobiology of Diabetic Complications: A Unifying Mechanism. Diabetes.

[6] Li J.-C., Shen X.-F., Meng X.-L. (2013). A traditional Chinese medicine JiuHuangLian (*Rhizoma coptidis* steamed with rice wine) reduces oxidative stress injury in type 2 diabetic rats. Food Chem. Toxicol..

[7] Yaribeygi H., Faghihi N., Mohammadi M.T., Sahebkar A. (2018). Effects of atorvastatin on myocardial oxidative and nitrosative stress in diabetic rats. Comp. Haematol. Int..

[8] Yaribeygi H., Mohammadi M.T., Sahebkar A. (2018). Crocin potentiates antioxidant defense system and improves oxidative damage in liver tissue in diabetic rats. Biomed. Pharmacother..

[9] Fakhruddin S., Alanazi W., Jackson K.E. (2017). Diabetes-Induced Reactive Oxygen Species: Mechanism of Their Generation and Role in Renal Injury. J. Diabetes Res..

[10] Lin X.-P., Ai W., Li M., Zhou X.-F., Liao S.-R., Wang J.-F., Liu J., Yang B., Liu Y.-H. (2019). Collacyclumines A–D from the endophytic fungus Colletotrichum salsolae SCSIO 41021 isolated from the mangrove *Kandelia candel*. Phytochemistry.

[11] Torres-Mendoza D., Ortega H.E., Cubilla-Rios L. (2020). Patents on endophytic fungi related to secondary metabolites and biotrans-formation applications. J. Fungi.

[12] Ortega H.E., Torres-Mendoza D., Caballero E. Z., Cubilla-Rios L. (2021). Structurally Uncommon Secondary Metabolites Derived from Endophytic Fungi. J. Fungi.

[13] El-Hawary S.S., Moawad A.S., Bahr H.S., Abdelmohsen U.R., Mohammed R. (2020). Natural product diversity from the endophytic fungi of the genus *Aspergillus*. RSC Adv..

[14] Koul M., Singh S. (2017). Penicillium spp.: Prolific producer for harnessing cytotoxic secondary metabolites. Anticancer. Drugs.

[15] de Carvalho A.C., Ogawa C.Y., Rodrigues L.D.C., de Medeiros L.S., Veiga T.A.M. (2021). *Penicillium* genus as a source for anti-leukemia compounds: An overview from 1984 to 2020. Leuk. Lymphoma.

[16] Mei R.-Q., Wang B., Zeng W.-N., Huang G.-L., Chen G.-Y., Zheng C.-J. (2021). Bioactive isocoumarins isolated from a mangrove-derived fungus *Penicillium* sp. MGP11. Nat. Prod. Res..

[17] Malika A., Ardalania H., Anama S., McNaira L.M., Kromphardtd K.J.K., Frandsend R.J.N., Franzyka H., Staerka D., Kongstad K.T. (2020). Antidiabetic xanthones with α-glucosidase inhibitory activities from an endophytic *Penicillium canescens*. Fitoterapia.

[18] Pan H.Q., Hu J.C., Wang S.J. (2017). A Plant Endophytic Fungus *Eupenicillium brefeldianum* F4a and Its Application. China Patent.

[19] Autschbach J. (2011). Time-Dependent Density Functional Theory for Calculating Origin-Independent Optical Rotation and Rotatory Strength Tensors. ChemPhysChem.

[20] Bruhn T., Schaumlöffel A., Hemberger Y., Bringmann G. (2013). SpecDis: Quantifying the Comparison of Calculated and Experimental Electronic Circular Dichroism Spectra. Chirality.

[21] Frisch M.J., Trucks G.W., Schlegel H.B., Scuseria G.E., Robb M.A., Cheeseman J.R., Scalmani G., Barone V., Mennucci B., Petersson G.A. (2009). Gaussian 09, Revision A.1.

[22] Bai Y., Chang J.-W., Xu Y., Cheng D., Liu H.-X., Zhao Y.-L., Yu Z.-G. (2016). Antioxidant and Myocardial Preservation Activities of Natural Phytochemicals from Mung Bean (*Vigna radiata* L.) Seeds. J. Agric. Food Chem..

[23] Bai Y., Xu Y., Chang J.-W., Wang X.-X., Zhao Y.-L., Yu Z.-G. (2016). Bioactives from stems and leaves of mung beans (*Vigna radiata* L.). J. Funct. Foods.

[24] Apostolidis E., Lee C.M. (2010). *In vitro* potential of *Ascophyllum nodosum* phenolic antioxidant-mediated *α*-glycosidase and *α*-amylase inhibition. J. Food Sci..

[25] Guo Z.-H., Niu X.-L., Xiao T., Lu J.-J., Li W., Zhao Y.-Q. (2015). Chemical profile and inhibition of *α*-glycosidase and protein tyrosine phosphatase 1B (PTP1B) activities by flavonoids from licorice (*Glycyrrhiza uralensis* Fisch). J. Funct. Foods.

[26] Jouda J.B., Kusari S., Lamshöft M., Talontsi F.M., Meli C.D., Wandji J., Spiteller M. (2014). Penialidins A-C with strong antibacterial activities from *Penicillium* sp., an endophytic fungus harboring leaves of *Garcinia nobilis*. Fitoterapia.

[27] Cheng X.-W., Yu L.-Y., Wang Q.-Q., Ding W.-J., Chen Z., Ma Z.-J. (2018). New brefeldins and penialidins from marine fungus *Penicillium janthinellum* DT-F29. Nat. Prod. Res..

[28] Yuan C., Wang H.Y., Wu C.S., Jiao Y., Li M., Wang Y.Y., Wang S.Q., Zhao Z.T., Lou H.X. (2013). Austdiol, fulvic acid and citromy-cetin derivatives from an endolichenic fungus, *Myxotrichum* sp.. Phytochem. Lett..

[29] Liu K.-W., Li Z.-L., Pu S.-B., Xu D.-R., Zhou H.-H., Shen W.-B. (2014). Chemical Constituents of the Rhizome of *Typhonium giganteum*. Chem. Nat. Compd..

[30] Elsayed Y., Refaat J., Abdelmohsen U.R., Ahmed S., Fouad M.A. (2017). Rhodozepinone, a new antitrypanosomal azepino-diindole alkaloid from the marine sponge-derived bacterium *Rhodococcus* sp. UA13. Med. Chem. Res..

[31] Tokes A.L., Bognar R. (1977). Synthesis of spiro chroman-4-ones. Flavonoids Bioflavonoids. Proc. Hung. Bioflavonoid Symp..

[32] Fu J., Hu L.W., Shi Z., Sun W., Yue D., Wang Y., Ma X.P., Ren Z.H., Zuo Z.C., Peng G.N. (2019). Two metabolites isolated from endophytic fungus *Coniochaeta* sp. F-8 in *Ageratina adenophora* exhibit antioxidative activity and cytotoxicity. Nat. Prod. Res..

[33] Heacock P.M., Hertzler S.R., Williams J.A., Wolf W.B. (2005). Effects of a medical food containing an herbal *α*-glycosidase inhibitor on postprandial glycemia and insulinemia in healthy adults. J. Amer. Dietetic Assoc..

[34] Genovese M., Nesi I., Caselli A., Paoli P. (2021). Natural *α*-Glucosidase and Protein Tyrosine Phosphatase 1B Inhibitors: A Source of Scaffold Molecules for Synthesis of New Multitarget Antidiabetic Drugs. Molecules.

