# Supplementary material for: Novel Antioxidants and α-Glycosidase and Protein Tyrosine Phosphatase 1B Inhibitors from an Endophytic Fungus Penicillium brefeldianum F4a
Source: J Fungi (Basel). 2021 Oct 27;7(11):913. doi: 10.3390/jof7110913 (PMC8623047; doi:10.3390/jof7110913)

Supporting Information

Novel antidiabetic and antioxidant secondary metabolites from plant-associated endophytic fungus *Penicillium brefeldianum* F4a

Yan Bai[1], Ping Yi[2], Songya Zhang[3], Jiangchun Hu[1], and Huaqi Pan*[, 1]

[1]Institute of Applied Ecology, Chinese Academy of Sciences, Shenyang 110016, People′s Republic of China

[2]The Key Laboratory of Chemistry for Natural Product of Guizhou Province and Chinese Academy of Sciences, Guiyang 550002, People′s Republic of China

[3]CAS Key Laboratory of Quantitative Engineering Biology, Shenzhen Institute of Synthetic Biology, Shenzhen Institute of Advanced Technology, Chinese Academy of Sciences, Shenzhen 518055, People′s Republic of China

Address correspondence to: Huaqi Pan

E-mail: panhq@iae.ac.cn;

Tel: 86-24-83970386

Fax:86-24-83970300

List of Supporting Information

Figure S1. HRESIMS spectrum of compound **1**.

Figure S2. IR spectrum of compound **1**.

Figure S3. ^{1}H NMR (600 MHz, DMSO-d_6) spectrum of compound **1**.

Figure S4. ^{13}C NMR (150 MHz, DMSO-d_6) spectrum of compound **1**.

Figure S5. HSQC spectrum of compound **1**.

Figure S6. ^{1}H-^{13}C HMBC spectrum of compound **1**.

Figure S7. ^{1}H-^{1}H COSY spectrum of compound **1**.

Figure S8. HRESIMS spectrum of compound **2**.

Figure S9. IR spectrum of compound **2**.

Figure S10. ^{1}H NMR (600 MHz, DMSO-d_6) spectrum of compound **2**.

Figure S11. ^{13}C NMR (150 MHz, DMSO-d_6) spectrum of compound **2**.

Figure S12. HSQC spectrum of compound **2**.

Figure S13. ^{1}H-^{13}C HMBC spectrum of compound **2**.

Figure S14. HRESIMS spectrum of compound **3**.

Figure S15. IR spectrum of compound **3**.

Figure S16. ^{1}H NMR (600 MHz, DMSO-d_6) spectrum of compound **3**.

Figure S17. ^{13}C NMR (150 MHz, DMSO-d_6) spectrum of compound **3**.

Figure S18. HSQC spectrum of compound **3**.

Figure S19. ^{1}H-^{13}C HMBC spectrum of compound **3**.

Figure S20. ^{1}H-^{1}H COSY spectrum of compound **3**.

Figure S1. HRESIMS spectrum of compound **1**

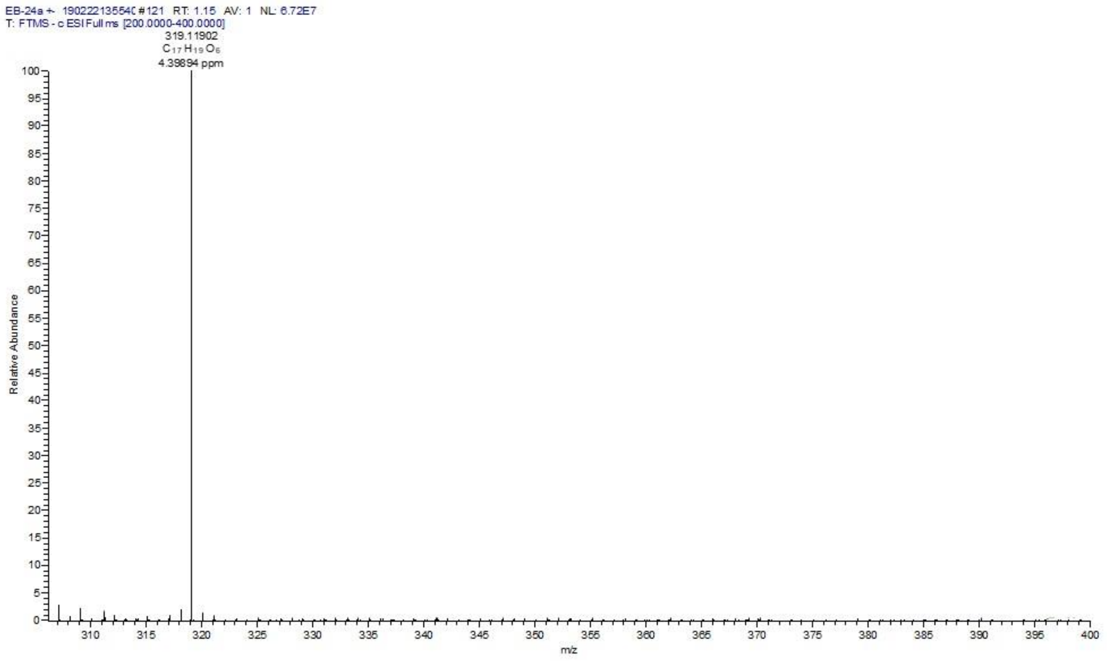

Figure S2. IR spectrum of compound **1**

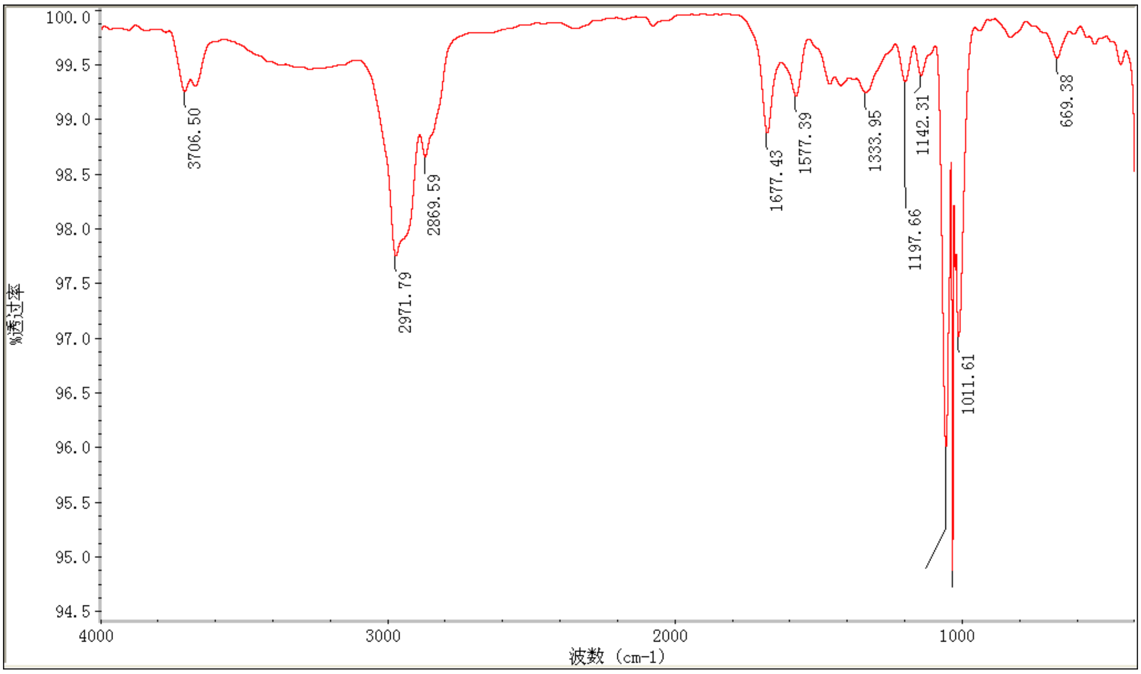

Figure S3. ^{1}H NMR (600 MHz, DMSO-d_6) spectrum of compound **1**

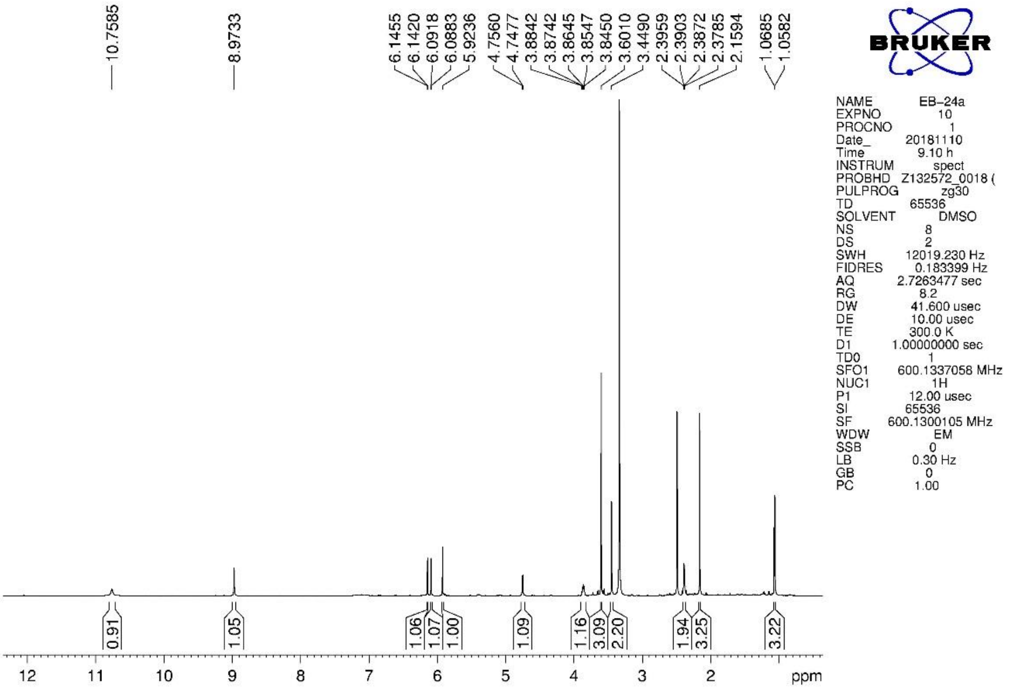

Figure S4. ^{13}C NMR (150 MHz, DMSO-d_6) spectrum of compound **1**

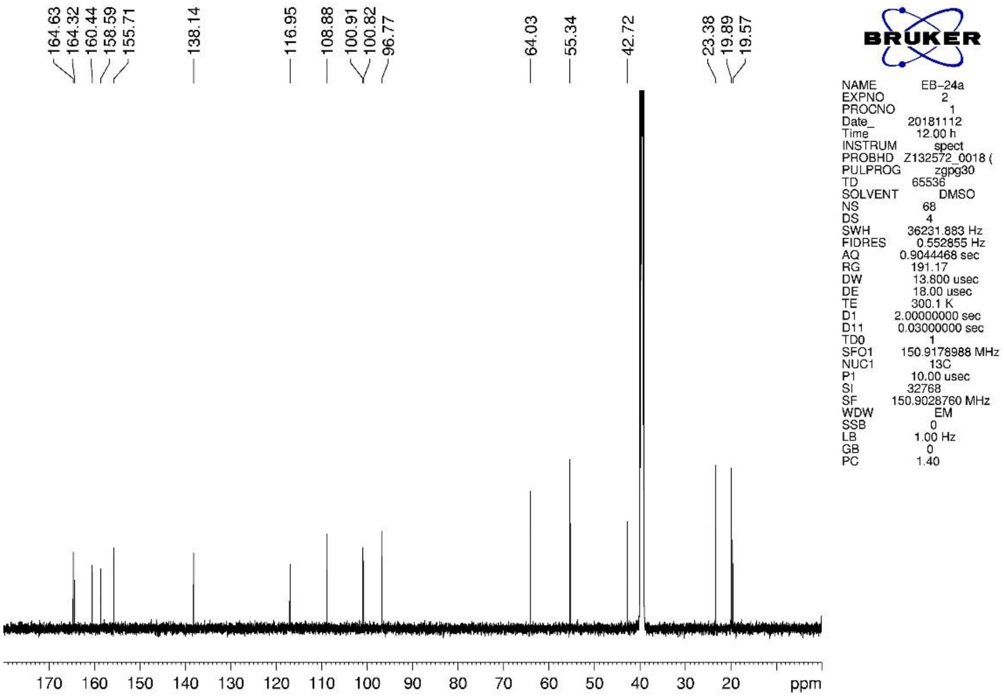

Figure S5. HSQC spectrum of compound **1**

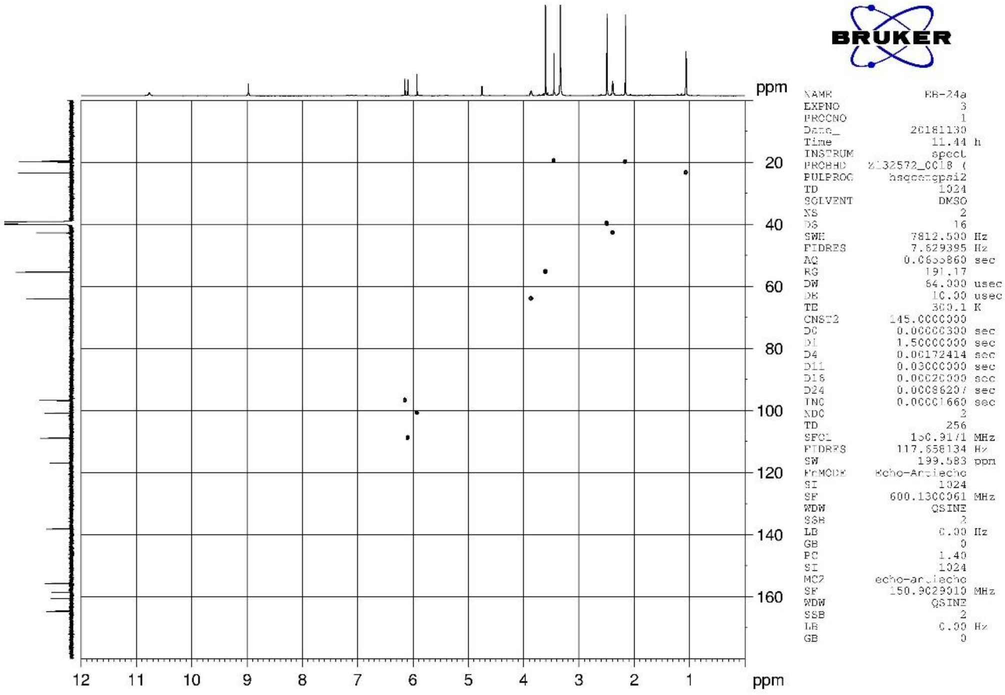

Figure S6. ^{1}H-^{13}C HMBC spectrum of compound **1**

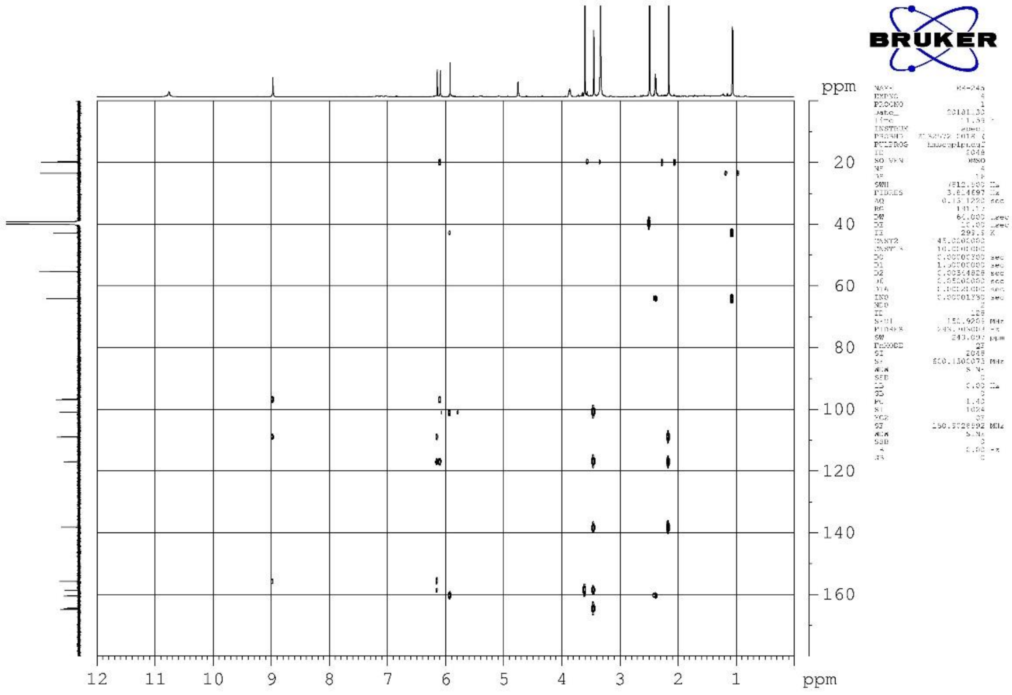

Figure S7. ^{1}H-^{1}H COSY spectrum of compound **1**

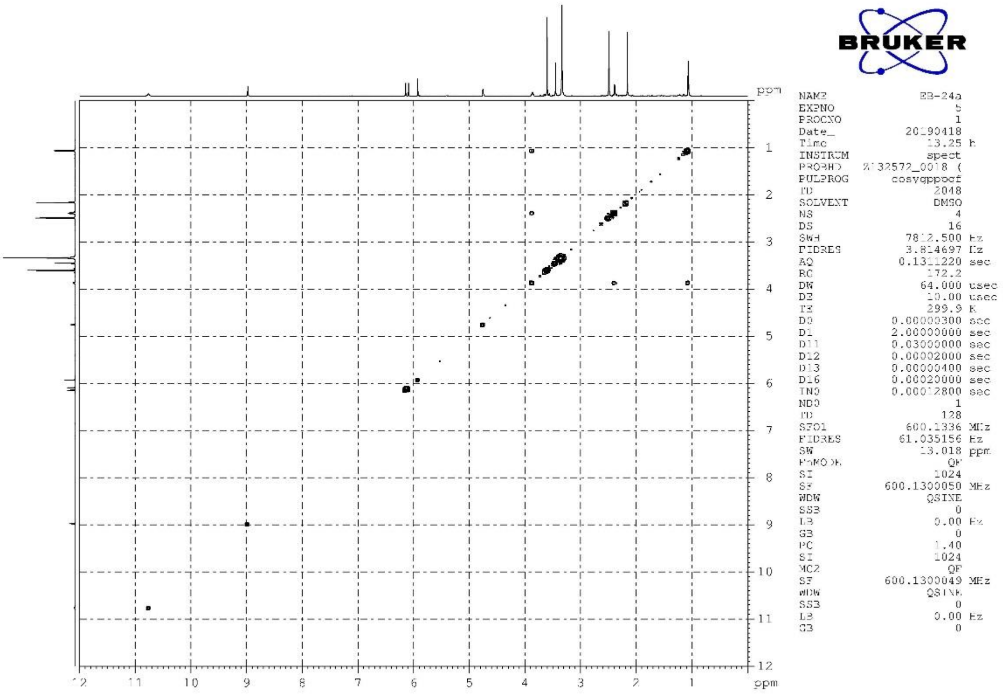

Figure S8. HRESIMS spectrum of compound **2**

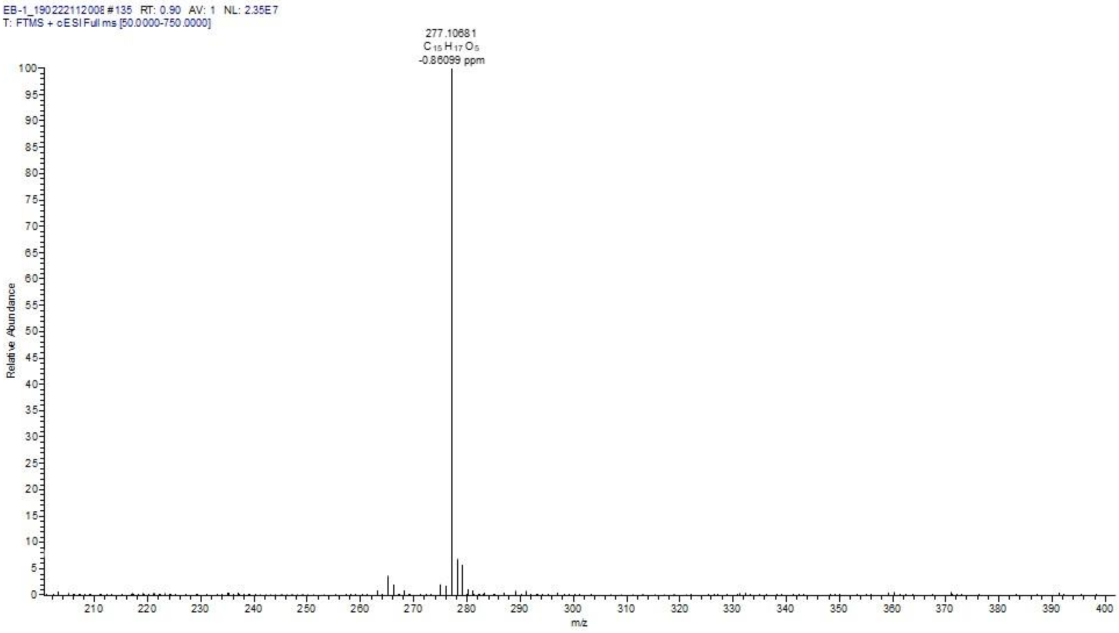

Figure S9. IR spectrum of compound **2**

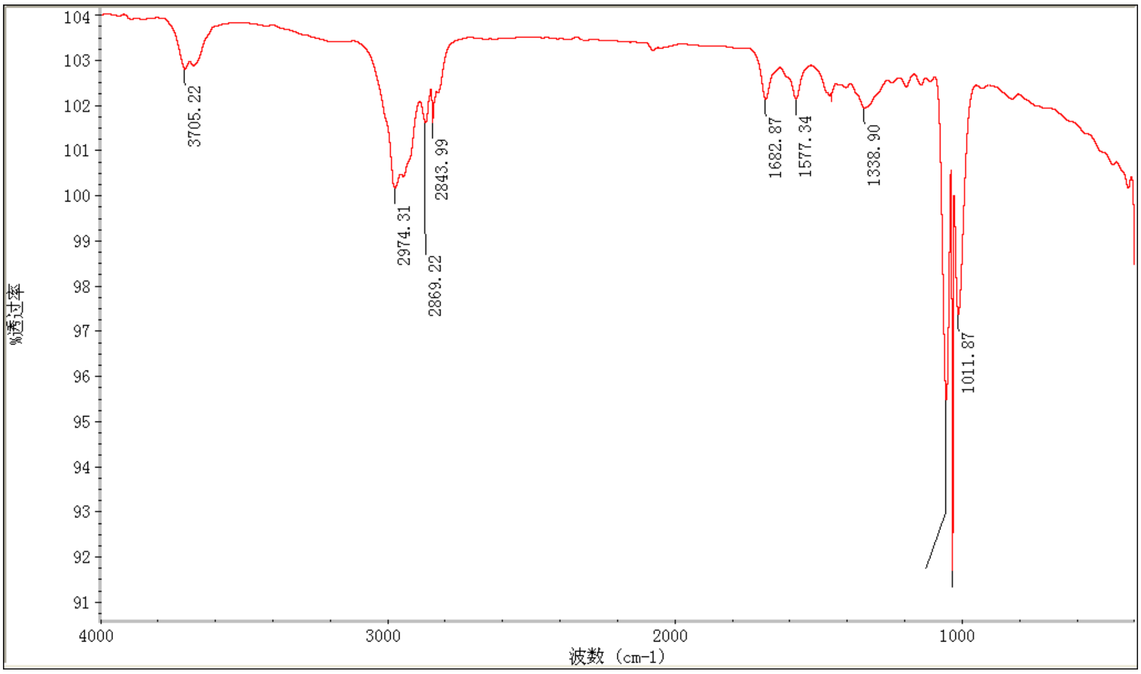

Figure S10. ^{1}H NMR (600 MHz, DMSO-d_6) spectrum of compound **2**

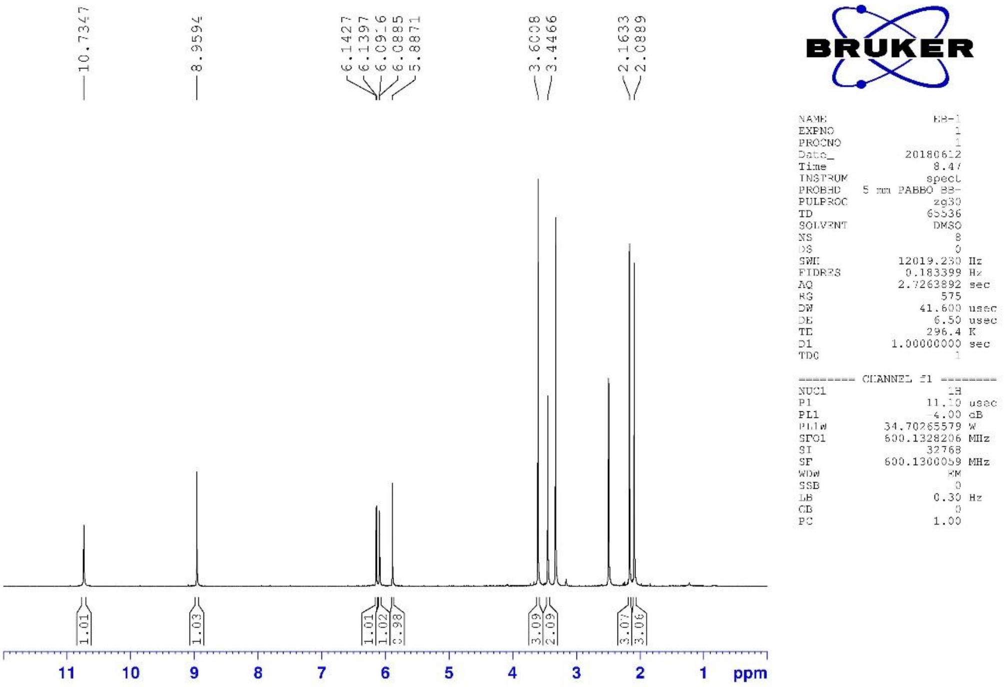

Figure S11. ^{13}C NMR (150 MHz, DMSO-d_6) spectrum of compound **2**

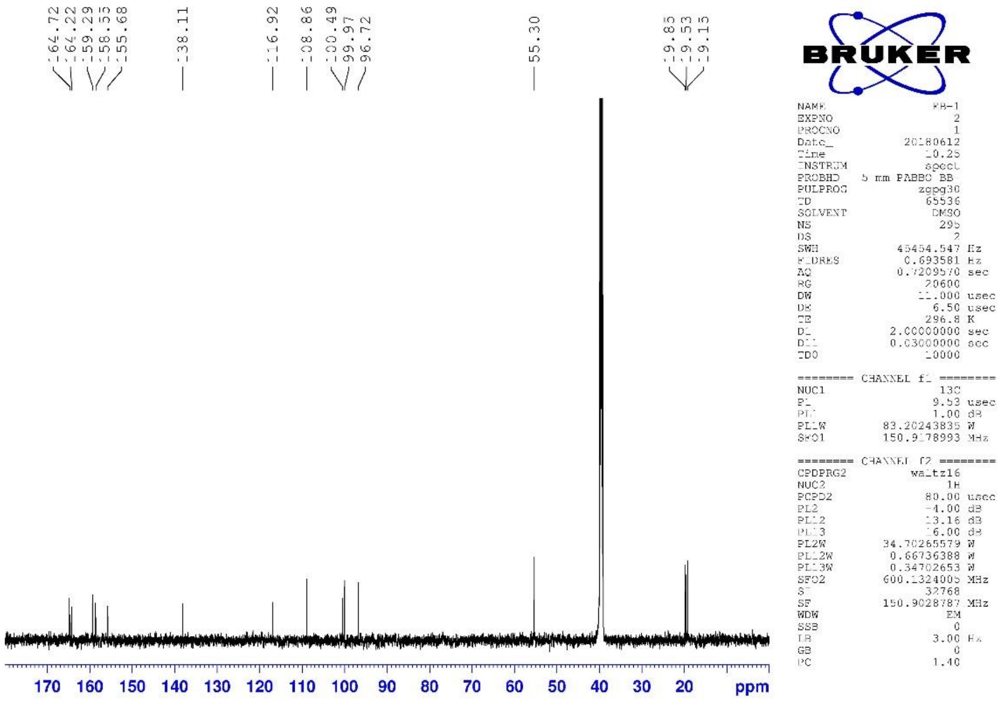

Figure S12. HSQC spectrum of compound **2**

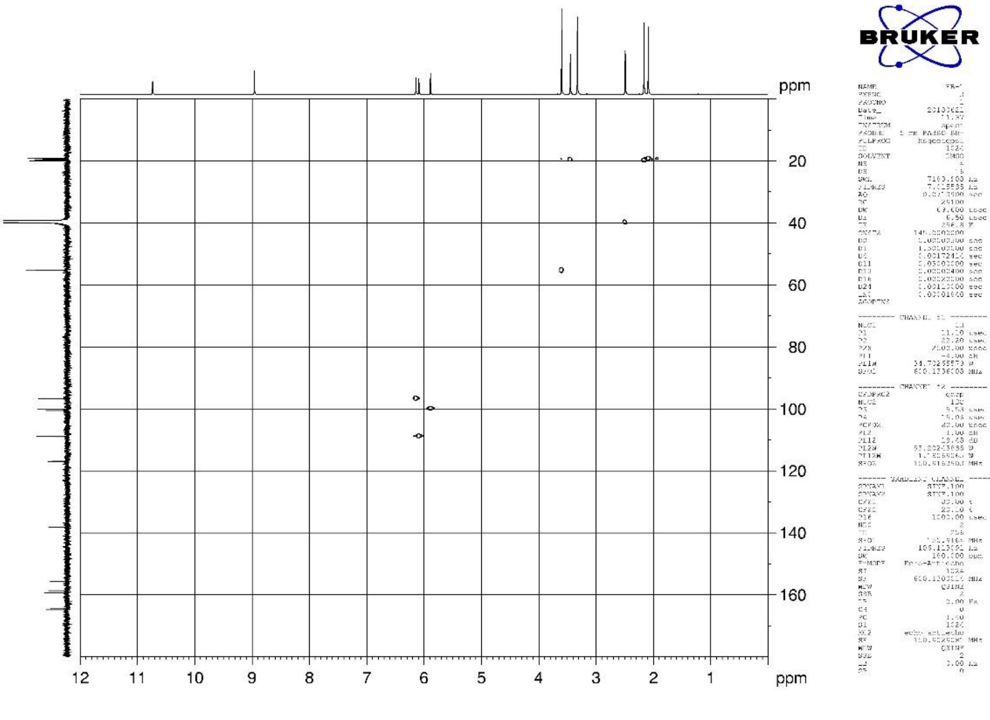

Figure S13. ^{1}H-^{13}C HMBC spectrum of compound **2**

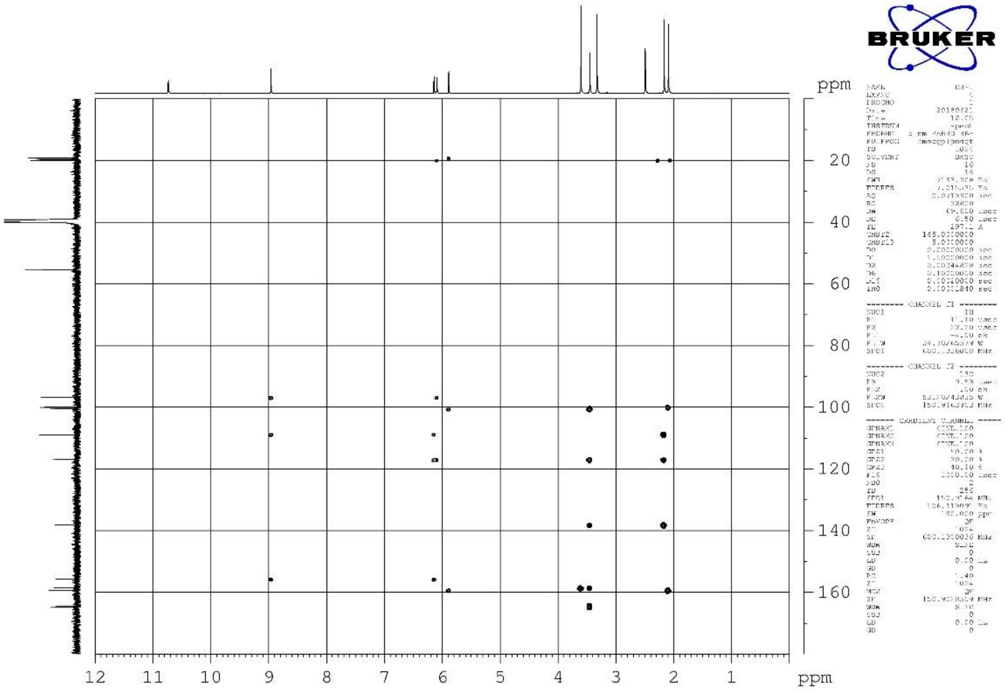

Figure S14. HRESIMS spectrum of compound **3**

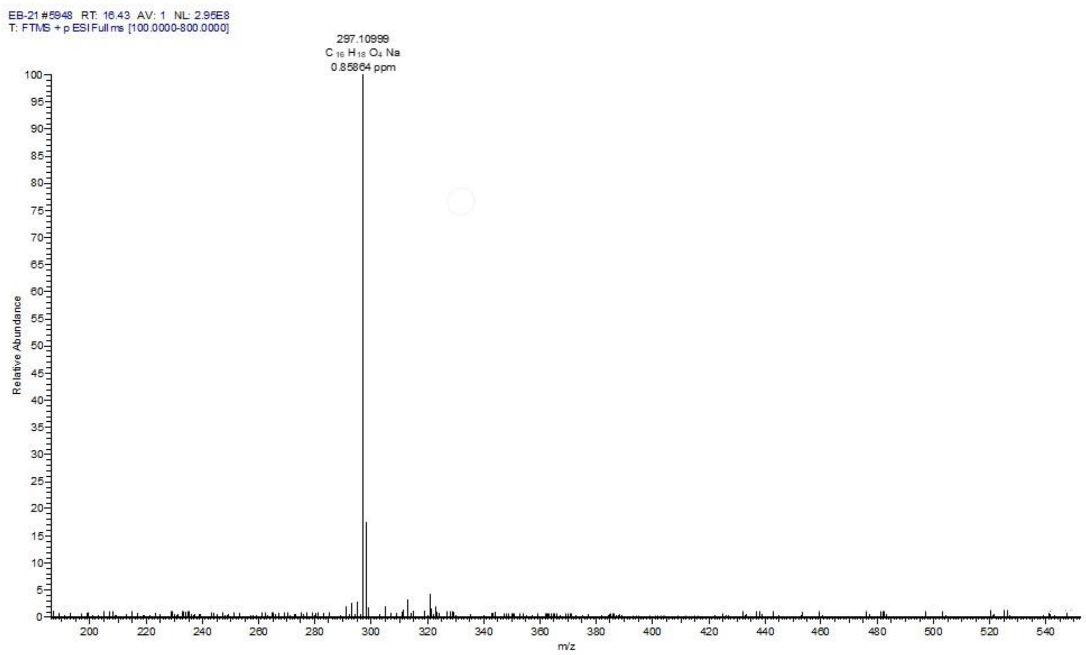

Figure S15. IR spectrum of compound **3**

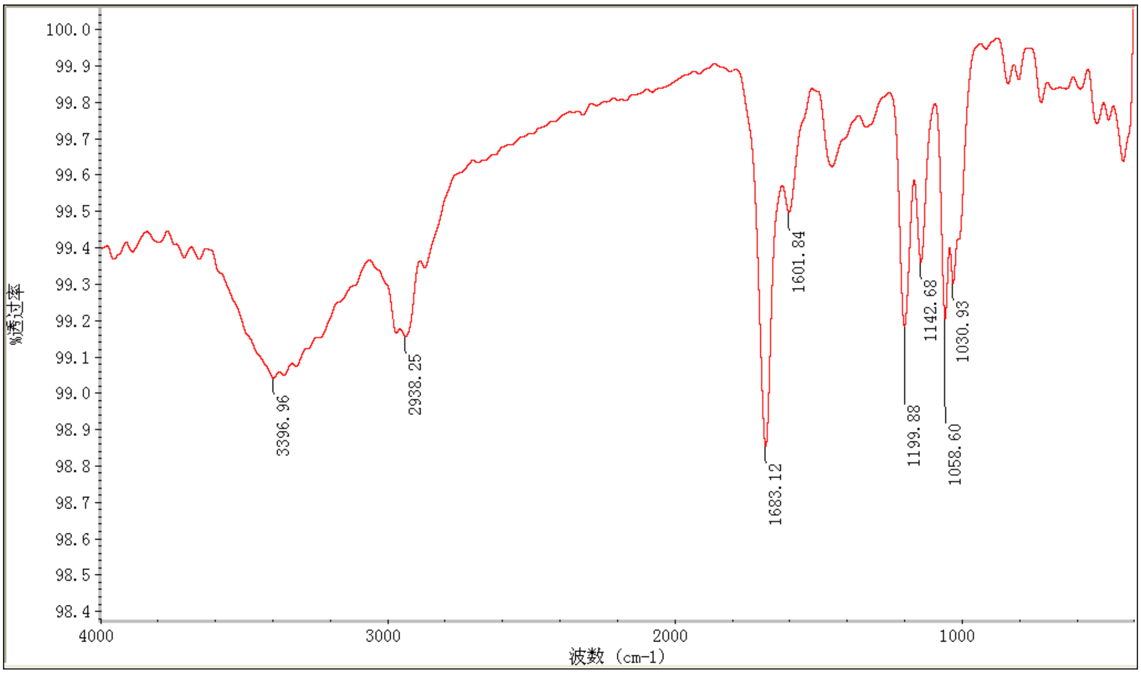

Figure S16. ^{1}H NMR (600 MHz, DMSO-d_6) spectrum of compound **3**

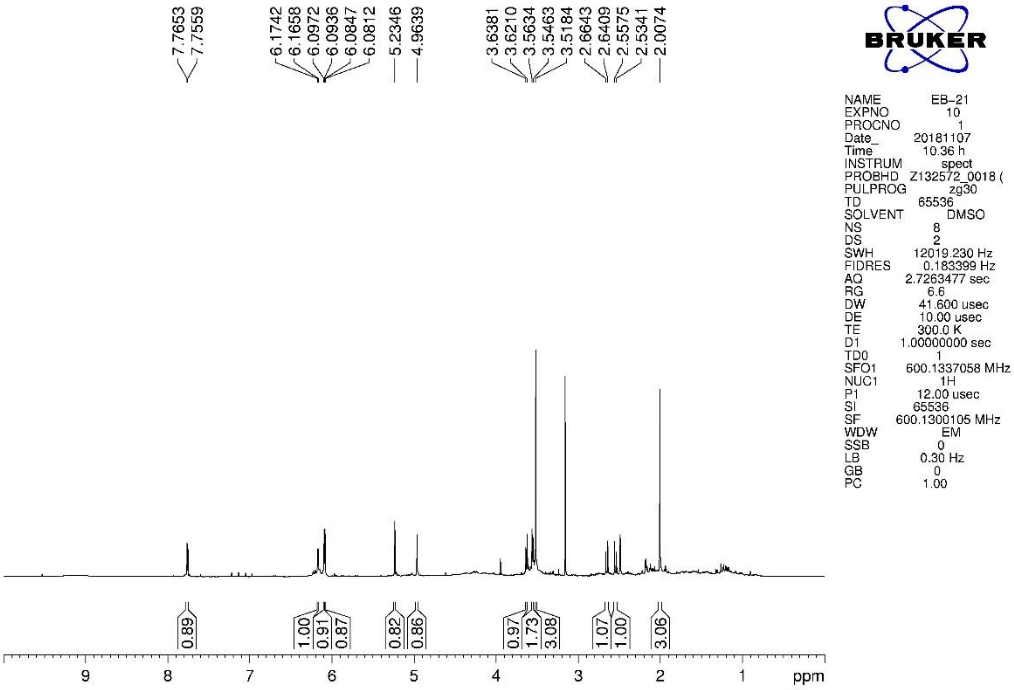

Figure S17. ^{13}C NMR (150 MHz, DMSO-d_6) spectrum of compound **3**

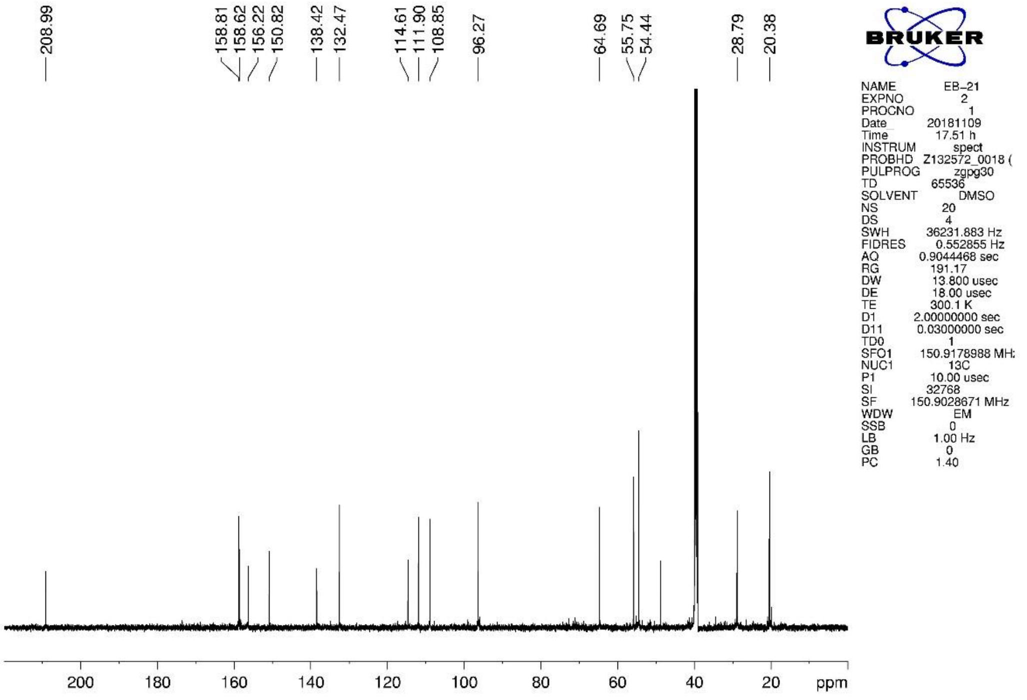

Figure S18. HSQC spectrum of compound **3**

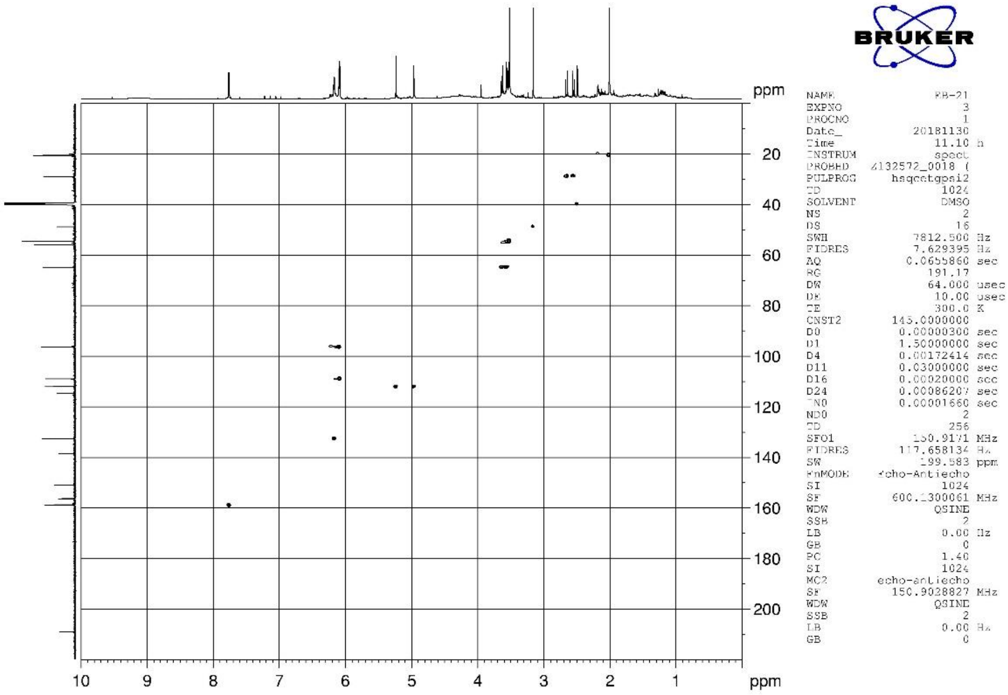

Figure S19. ^{1}H-^{13}C HMBC spectrum of compound **3**

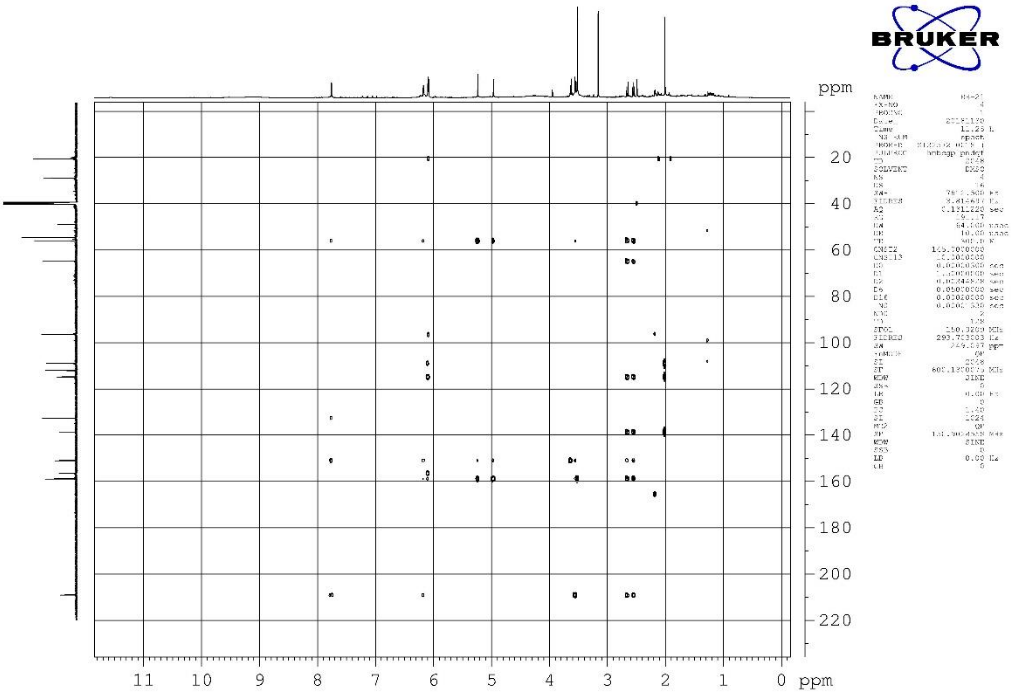

Figure S20. ^{1}H-^{1}H COSY spectrum of compound **3**

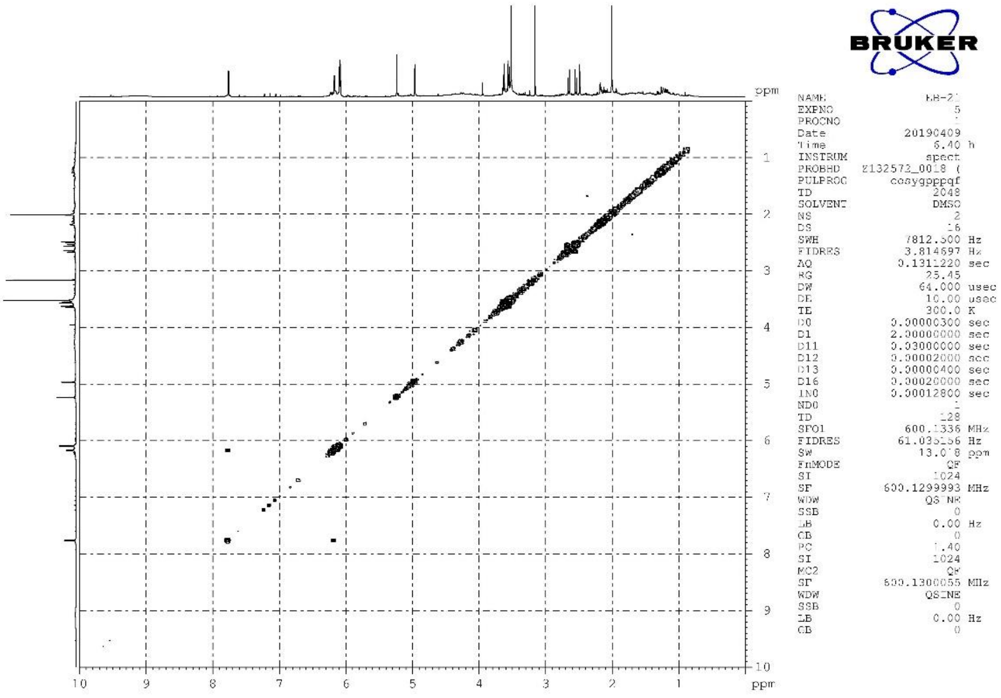

Supplement: Supplementary file 1 [file jof-07-00913-s001.zip › jof-1432188-supplementary.pdf]
